# Electrical Properties of Silver-Attached Amine Functionalized Carbon Black/Polyethylene Terephthalate Fibers Prepared by Melt-Spinning

**DOI:** 10.3390/polym11101611

**Published:** 2019-10-03

**Authors:** Hyun-Jung Choi, Damiro Ahn, Sohee Lee, Sang Young Yeo

**Affiliations:** 1 Technical Textile Materials R&D Group, Korea Institute of Industrial Technology, 143 Hanggaulro, Sangnok-gu, Ansan-si, Gyeonggi-do 15588, Korea; hjchoi@kitech.re.kr (H.-J.C.); ahndamiro94@kitech.re.kr (D.A.); 2 Department of Clothing and Textiles, Research Institute of Natural Science, Gyeongsang National University, 501 Jinju-dearo, Jinju-si, South Gyeongsang Province 52828, Korea; sohee.lee@gnu.ac.kr

**Keywords:** conductive fiber, melt-spinning, silver nanoparticle, carbon black, PET

## Abstract

In this study, amine functionalized carbon black (ABCB) was synthesized using 4-aminobenzoic acid in a phosphoric acid (PPA)/phosphorus pentoxide (P_2_O_5_) medium, and silver-attached carbon black (Ag-ABCB) was prepared by reducing AgNO_3_ with NaBH_4_ in the presence of ABCB in ethanol. Elemental, thermogravimetric, and Fourier transform-infrared analyses showed that carbon black (CB) had a well-functionalized 4-aminobenzoic acid. In addition, X-ray photoelectron spectroscopy and X-ray diffraction were used to examine the crystal structure of Ag nanoparticles. Conductive fibers were prepared by melt-spinning using ABCB, Ag-ABCB as a conductive filler, and polyethylene terephthalate (PET) as a polymer matrix. Results confirmed that the fiber that had Ag-ABCB as a conductive filler exhibited the best electrical conductivity. The dispersibility and morphology of the conductive filler in the PET matrix were confirmed through scanning electron microscopy analysis, and Ag-ABCB was the most uniformly dispersed filler in the PET matrix, with good structure.

## 1. Introduction

As technology continues to improve, new conductive materials for thinner, lighter, and more portable displays are increasingly in demand. The applications on the conductivity range, and materials with electrical conductivity can be applied to electrostatic discharge products (1–10^11^ Ohm/sq) [1,2] and electromagnetic interference products (EMI: 1–10^−2^ Ohm/sq) [3,4]. As the convergence of fiber and information technology (IT) has accelerated, the various wearable device industry has developed materials with excellent conductivity and the capability to transmit electric signals. For wearable materials including films [5,6], fibers [7,8], and fabrics [9,10], flexibility is crucial to the technical textile industry. Methods to produce conductive fibers include solutions spinning [11], metal coating [12], electrospinning [13,14], bubbfil spinning [15], and melt-spinning [2,16]. Except for melt-spinning, these processes are not suitable for mass production due to high costs and complex manufacturing. Melt-spinning, however, is a cheap mass-production method. In addition, conductive fibers produced by melt-spinning exhibit excellent fiber mechanical strength compared to the other four methods. Despite these advantages, melt-spinning has not been widely used in industry to produce conductive fibers, because it is difficult to control the mixing ratio, screw speed, feed rate and dispersion conditions. Therefore, research on identifying optimal process conditions is still required.

When producing conductive fibers, there are limiting factors such as tensile strength, washing ability, reliability, and the stability of metal conductive or conductive polymer coated yarn. As such, it is necessary to add conductive fillers such as carbon black (CB) [2,14], carbon nanotubes [11,16,17], graphite [18,19], and metal powders [20] to fabricate conductive fibers. CB is one of the most commonly-used fillers for plastics. When CB is mixed with the polymer matrix, mechanical strength improves and the specific surface area increases, resulting in better electric conductivity. The dispersibility of the filler used in melt-spinning is very important, and CB has better spinnability than other carbon fillers due to its small size. Recently, CB/polymer conductive composites have been studied in various fields. However, most studies have been limited to the production of polymeric resins and simple physical composites using the original CB. It is therefore necessary to study filler modification to improve dispersion and conductivity in the polymer resin. To modify the filler, Iijima devised a carbon nanotube (CNT) [21], and a method to modify it in sulfuric acid and nitric acid has been widely used [22,23]. 

A polyphosphoric acid (PPA)/phosphorus pentoxide (P_2_O_5_) reaction medium, which efficiently functionalizes CB while retaining its inherent properties, is well known because it causes inherent surface damage and roughness [24,25,26,27,28]. PPA has sufficient acidity to provide protons to the surface of CB, creating an atmosphere in which the substitution reaction can take place by bonding hydrogen to the surface of hydrogen-free CB. The use of PPA can also lead to electrophilic substitution reactions in the absence of electrons, such as carbon nanomaterials. This was confirmed through reactions with single-walled carbon nanotubes (SWNTs) [28], multi-walled carbon nanotubes (MWNTs) [25,26], and vapor-grown carbon nanofibers (VGCNFs) [27]. A mild PPA/P_2_O_5_ medium is not only an efficient reaction medium but can also prevent re-aggregation and improve dispersion.

Silver (Ag) exhibits the largest electrical and thermal conductivities among all metals [29]. Therefore, functional nanocomposites with desired properties can be tailored by incorporating Ag nanoparticles into polymers [30]. Ag nanoparticles with a high surface area possess remarkable structural, electronic and thermal properties. Therefore, electrical conductivity can be improved by attaching Ag nanoparticles. In this study, we fabricated fibers using four different types of conductive fillers to the polyethylene terephthalate (PET) matrix through the melt-spinning method and investigated the electrical properties of these fibers. To do so, 4-aminobenzoyl functionalized carbon black (ABCB) was synthesized using a Friedel–Crafts acylation reaction in a PPA 88% assay/P_2_O_5_ reaction medium, which can efficiently introduce organic functional groups into CB. Silver-attached carbon black (Ag-ABCB) with Ag nanoparticles attached to ABCB was then prepared. The electrical conductivity of fibers using ABCB, Ag-ABCB, commercialized CB, and Ag/CB as conductive fillers was investigated. The functionalization of CB was characterized by elemental analysis (EA), thermogravimetric analysis (TGA), and Fourier transform-infrared (FT-IR) analysis. The crystal structure of Ag nanoparticles was investigated using X-ray photoelectron spectroscopy (XPS) and X-ray diffraction (XRD).

## 2. Experiments

### 2.1. Materials

4-Aminobnezoic acid, PPA (83% P_2_O_5_ assay) and P_2_O_5_ were all obtained from Sigma Aldrich Chemical Inc., St. Louis, MO, USA, and used without any treatment. Methanol was used as-received from Daejung Chemmical & Matals Co. Ltd., Siheung, Korea with a purity >99.5%. CB (Ketjen Black EC600JD^®^, Amsterdam, The Netherlands) with a surface area of 1270 m^2^/g and pore volume of 4.8–5.1 mL/g (as determined by dibutyl phthalate absorption) was purchased from AkzoNobel. The Ag powder NP-S80 (NTbase, Yongin, Korea) was used. The polymer matrix resin was a commercial grade polymer, and polyethylene terephthalate (PET; JSD588^®^, Huvis Co., Seoul, Korea) with intrinsic viscosity of 0.64 and melt flow rate of 33.6 was used. All materials were used after vacuum drying at 120 °C for 6 h before extrusion for moisture removal.

### 2.2. Methods

#### 2.2.1. Synthesis of ABCB

4-Aminobenzoic acid (ABAc, 20 g), CB (20 g), PPA (83% P_2_O_5_ assay; 1000 g), and P_2_O_5_ (250 g) were placed in a 2 L four-neck reactor flask equipped with a high-torque mechanical stirrer in addition to nitrogen inlet and outlet taps. The reaction mixture was heated to 50 and 100 °C for 1 h each to sufficiently disperse CB in the medium. Subsequently, the mixture temperature increased to 130 °C and was further stirred for 48 h. At the end of the reaction, the mixture viscosity slowly increased and excess H_2_O was poured into the final product. The resulting precipitate was poured into distilled water, and the product was collected by filtration. The product was Soxhlet-extracted with H_2_O to remove any residual reaction medium for 3 days, and then with methanol for another 3 days to remove unreacted 4-aminobenzoic acid and possible impurities. Finally, samples were freeze-dried under reduced pressure (5 mmHg) and the final black powder (yield: 32.64 g, 86.99%) was collected. Anal. Calcd. for C_15.81_H_6_ON (calculation based on % yield): C 82.98%; H 2.36%; O 6.22%; N 5.44% and found C 83.49%; H 2.03%; O 9.98%; N 4.54%.

#### 2.2.2. Synthesis of Ag-ABCB

The Ag-ABCB nanocomposite was prepared using an established method [31]. First, the ABCB powder (10 g) was dispersed in 2 L of ethanol for 1 h under strong stirring. AgNO_3_ (0.1 M), a silver precursor, was added to the Ag-ABCB nanocomposite mixture in an ice bath, and black suspension became light yellowish. Next, an ice-cold aqueous NaBH_4_ (0.2 M) solution was added dropwise to the mixture under vigorous magnetic stirring for 2 h. The solution turned dark yellow immediately after adding the NaBH_4_ solution, indicating that the NaBH_4_ reduced the ionic silver and stabilized the silver nanoparticles that formed [32]. After the reaction was complete, the final product was washed with water and ethanol several times, then collected by vacuum filtration and freeze-dried under a reduced pressure (0.5 mmHg).

The chemical reaction was the NaBH_4_ reduction of AgNO_3_:

(1) $$AgNO_{3}+NaBH_{4}\;\to Ag+\frac{1}{2}H_{2}+\frac{1}{2}B_{2}H_{6}+NaNO_{3}$$

### 2.3. Processing

**Figure 1:**
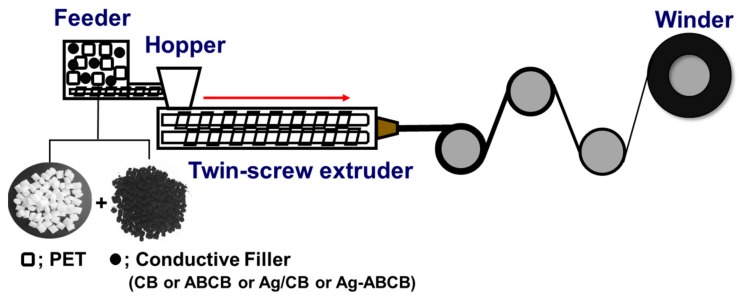
Schematic presentation of the fiber preparation by using melt-spinning process. Conductive fillers used to make conductive fibers are carbon black (CB), amine functionalized carbon black (ABCB), silver-attached carbon black (Ag-ABCB) and Ag/CB.

A twin-screw extruder (BA-11, Bautek Co., Pocheon, Korea) with a screw diameter of 11 mm and a 3 mm nozzle with a length-to-diameter ratio (L/D) of 3.64 was used to prepare a conductive fiber with conductive filler dispersed in the PET matrix. In this study, PET was used as a polymer matrix. Four conductive fillers were used: pristine CB, chemically functionalized ABCB, Ag-ABCB, and physically mixed Ag power and CB (Ag/CB) (Ag/CB = 10/90 wt%). Amounts of 0.5, 1, 2, 3, 4, and 5 wt% of CB, ABCB, Ag-ABCB, and Ag/CB were mixed with each PET polymer matrix and then conductive filler was injected into the hopper of an extruder. The temperature, screw rotation speed, and feeder speed during extrusion are shown in Table 1. An amount of 3 wt% was wound up at high speed, but 4 wt% was difficult to fiberize and thus was wound up manually (Figure 1, Table 1). Sample names are shown as conductive filler/PET_X, where X is the conductive filler content.

### 2.4. Characterizations

EA was completed using Flash 2000 (ThermoScientific, Waltham, MA, USA). FT-IR analysis was performed using Perkin-Elmer, USA. All samples were mixed with dried KBr and pressed to form semitransparent pellets. TGA was measured using a Q500 (TA Instrument, USA) from room temperature up to 800 °C at a heating rate of 10 °C/min in air. The XRD powder pattern was measured using a D/MAX Ultima II diffractometer with a theta-theta goniometer (Cu Kα radiation, λ = 1.54056 Å, Rigaku, Tokyo, Japan) at 40 kV, at a scan rate of 2°/min for scan angles 2θ = 10–80°. XPS spectra were obtained from an X-ray photoelectron spectrometer using K-Alpha (Thermo Fisher Scientific, Waltham, MA, USA).

The electrical conductivity of all conductive filler/PET composite fibers was measured using a Keithley 6517B^®^ two-point probe high-resistance meter (Keithely Instruments, Inc, Cleveland, OH, USA). All measurements were performed in accordance with the ASTM-D-257 standard resistance measuring method. To measure resistance, the sample was fixed with a silver paste at a distance of 1 cm on an insulating glass plate, and the resistance was measured after applying 100 V for 1 min to stabilize the sample. The electrical conductivity was calculated by the following Equation [33]:

(2) $$S_{2-point}\left(\frac{S}{cm}\right)=\frac{1}{V}\;\times \frac{l}{S}\;\times \frac{1}{N}$$

where *V* is voltage, *l* is length, *S* is the cross-sectional area of the fiber, and *N* is the number of filaments.

Field emission-scanning electron microscopy (FE-SEM; SU8000, Hitachi Ltd. Tokyo, Japan) was used to confirm the synthesized conductive filler morphologies and degree of dispersion in the PET matrix. All samples were cut after pretreatment with liquid nitrogen.

## 3. Results and Discussion

### 3.1. Characteristics of ABCB and Ag-ABCB Nanocomposites

To functionalize a carbon material, a carboxyl group must be produced on its surface by treating it with an acid such as a sulfuric acid/nitric acid mixture, and then reacting it with another reactive group. This acid treatment method easily produces a carboxyl group but causes many defects on the carbon material surface [23,34]. Unlike the sulfuric acid/nitric acid treatment method, the “direct” Friedel–Crafts acylation reaction can be successfully completed with only a small amount of impurities on the carbon material surface. This method uses a weak acid PPA/P_2_O_5_ medium to introduce the functional group into the CB surface, while minimizing the CB surface damage. 

**Figure 2:**
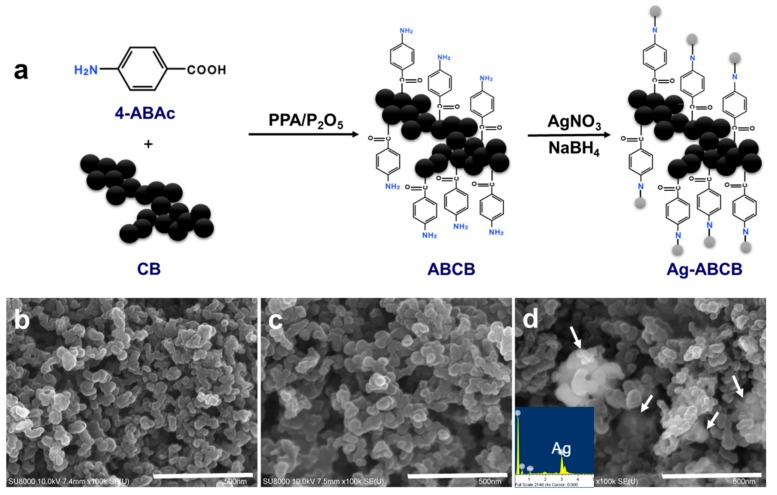
(**a**) Schematic presentation of the synthesis of ABCB with in PPA/P_2_O_5_ medium and Ag-ABCB conductive fillers. SEM images of (**b**) CB; (**c**) ABCB; (**d**) Ag-ABCB (Inset is EDX of ABCB). Scale bar is 0.5 μm.

Figure 2a shows the synthesis of ABCB by reacting 4-aminobenzoic acid with CB, using the "direct" Friedel–Crafts acylation reaction. This method was first introduced by Baek et al. [24]. When the ABCB is synthesized by the reaction shown in Figure 2a, the amine group (–NH_2_) is present. Here, AgNO_3_ was used as a metal salt, and subsequently an Ag attached conductive filler (Ag-ABCB) was reduced by NaBH_4_. ABCB and Ag-ABCB were used as the conductive filler, while the physically-mixed Ag power/CB mixture (Ag/CB) and commercially available pristine CB were used as comparative conductive fillers to examine electrical conductivities and morphologies.

EA of the functionalized ABCB showed that the theoretical value of CB before reforming was 100.0%, while hydrogen and oxygen content were both 0.0%. However, the analyzed carbon was 97.3%, with a hydrogen and oxygen content of 0.4% and 0.7%, respectively. Because the CB purity was approximately 99.9%, there were differences between the theoretical and analytical values. The calculated carbon content of functionalized ABCB was 82.98%, with hydrogen content of 2.36%, oxygen content of 6.22%, and nitrogen content of 5.44%. However, the analyzed carbon content was 83.49%, with hydrogen, oxygen, and nitrogen content of 2.03%, 9.98%, and 4.54%, respectively. The following slight differences between calculated and analyzed values were due to the purity of CB as the starting material: carbon content: 0.51%; hydrogen content: 0.36%; oxygen content: 3.76%; nitrogen content: 0.90%. 

SEM images showed gradual changes during the reaction of pristine CB, ABCB, and Ag-ABCB, with an agglomerate CB and ABCB particle size of approximately 60–70 and 70–80 nm, respectively (Figure 2c). These results support the functionalization of the 4-aminobenzoyl group. In addition, the bright and white parts indicated by the arrows show that the partially-aggregated silver nanoparticles were well loaded to the ABCB surface (Figure 2d). The Figure 2d inset shows clear Ag peaks from Ag-ABCB nanocomposites, visually emphasizing that the Ag nanoparticles were effectively attached to the ABCB surface.

**Figure 3:**
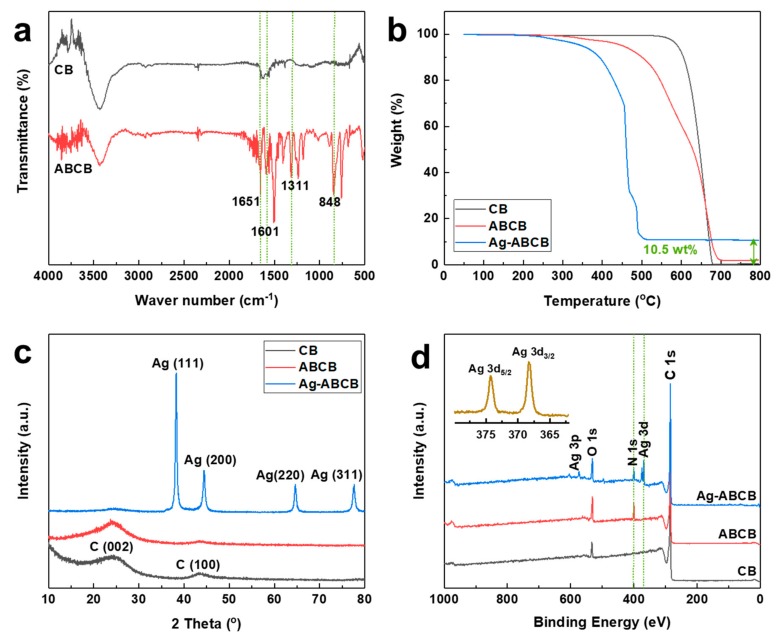
(**a**) FT-IR spectrum of samples; (**b**) TGA thermograms of the samples using a heating rate of 10 °C/min in air; (**c**) X-ray powder diffraction patterns of samples; (**d**) XPS spectra of CB, ABCB, and Ag-ABCB; High resolution XPS spectra of (inset: Ag3d doublet).

FT-IR spectroscopy is a very important technique used to study the functionalization of carbon materials. FT-IR was used to confirm that the 4-aminobenzoyl group was efficiently functionalized on the CB surface (Figure 3a). Pristine CB did not exhibit any specific peak, while ABCB showed a primary amine (1603 cm^−1^). The CN stretching band of the aromatic amine was 1311 cm^−1^, and the NH out of the plane bend was 848 cm^−1^. ABCB also exhibited an aromatic carbonyl (C=O) peak at 1651 cm^−1^ [25]. It was therefore confirmed that the 4-aminobenzoyl group was successfully functionalized on the CB surface. 

The degree of functionalization and amount of Ag loaded were quantitatively estimated by TGA. Figure 3b shows the thermal stability of CB, ABCB, and Ag-ABCB, with the TGA studied from room temperature up to 800 °C at a heating rate of 10 °C/min in air. The weight of pristine CB did not change up to nearly 600 °C, but a stepwise weight loss occurred in the functionalized ABCB. The initial weight loss at 548 °C was attributed to the 4-aminobenzoyl group covalently bonded to the CB edge. The organic moiety on the ABCB could be estimated from the weight loss occurring at 548 °C and was approximately 40 wt% of the AB moiety. The pyrolysis that occurred at 664 °C was attributed to CB. For Ag-ABCB in the air, the char yield was approximately 10.5 wt% at 800 °C, while CB and ABCB had values close to 0%. These results were attributable to the Ag nanoparticle. Overall, TGA results showed that the 4-aminobenzoyl group was covalently attached to the CB surface and the silver nanoparticles were well-attached to the functionalized ABCB. 

Figure 3c shows the XRD pattern of the pure CB, ABCB, and Ag-ABCB nanocomposites. The peaks at 24.2 and 43.3° were attributed to the C (002) and C (100) planes of pristine CB, which were crystalline graphite-like material [35]. Meanwhile, ABCB had a peak that was not significantly different from the pristine CB peak. In addition, characteristic diffraction peaks observed at 38.1, 44.3, 64.7, and 77.5° were attributed to the typical crystallographic planes of Ag (111), Ag (200), Ag (220), and Ag (311) [36]. Ag nanoparticles were strongly bound to the CB surface by amino functionalization, while binding energy between the Ag atom and pure CB was very weak. Therefore, Ag nanoparticles bonded strongly to the surface of Ag-ABCB.

XPS analysis confirmed that the well-functionalized CB and Ag attached on the ABCB, exemplified by the presence of C, O, N, and Ag in Ag-ABCB (Figure 3d). Pristine CB exhibited a strong C 1s peak at 284.5 eV, and an O 1s peak at 532.4 eV [37]. The oxygen element was consistent with the EA findings, and the purity of pristine CB seemed to be influential. For the 4-aminobenzoyl group, the N 1s peak was found at 399.8 eV [38] in ABCB, and the intensity of the O 1s peak increased, suggesting that the 4-aminobenzoyl group was successfully functionalized. After attaching the silver nanoparticles, a new peak (Ag 3d) was observed. Ag 3d_5/2_ (374.3 eV) and Ag 3d_3/2_ (268.3 eV) both exhibited peaks. These peaks were a typical form of Ag^0^ [39] and strong indicators of the presence of Ag particles on the ABCB surface.

### 3.2. Electrical Properties of Conductive Filler/PET Fiber

Conductive filler/PET fiber was fabricated by placing the CB, ABCB, Ag-ABCB, and Ag/CB into the PET matrix. The temperatures of the six zones were controlled with a twin-screw extruder, and the screw speed and feeder speed were adjusted for each composite (Table 1).

**Figure 4:**
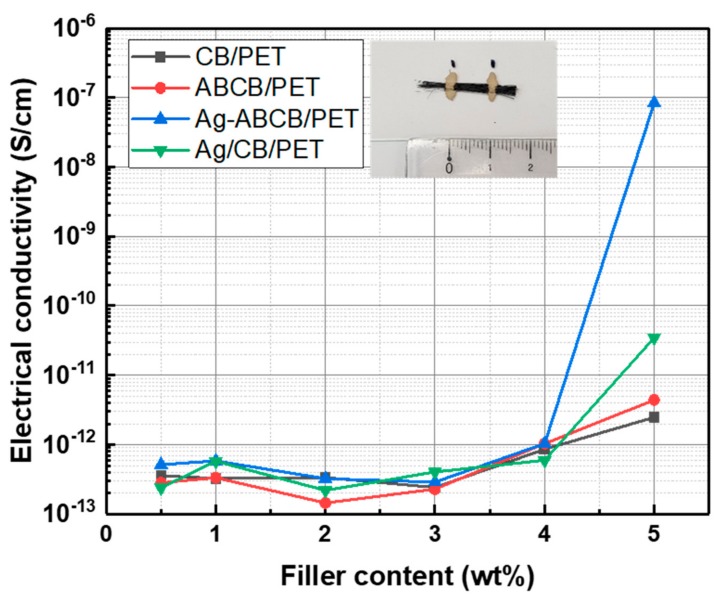
Electrical conductivity of CB/PET, ABCB/PET, Ag-ABCB/PET, and Ag/CB/PET composite fibers.

To investigate the correlation of electrical conductivity with the type of conductive filler in the fiber, four kinds of conductive fillers (CB, ABCB, Ag-ABCB, Ag/CB) were used. The filaments were placed and fixed with silver paste at intervals of 1 cm (Figure 4 inset). The electrical conductivity of four kinds of fibers was measured a total of 10 times and the average value of these electrical conductivity was used in Figure 4. As shown in Figure 4, the electrical conductivity of the conductive filler/polymer composite fiber was not significantly increased at low CB content, and the electrical conductivity of the conductive composite at the weight ratio of the conductive filler occurred in the range of 4–5 wt%. The electrical conductivity of CB/PET5 was 2.48 × 10^−12^ S/cm, ABCB/PET5 was 4.41 × 10^−12^ S/cm, Ag-ABCB/PET5 was 8.41 × 10^−8^ S/cm, and Ag/CB/PET5 was 3.48 × 10^−11^ S/cm. These results showed that the Ag-ABCB/PET5 fiber had an electrical conductivity approximately 3 × 10^4^ times higher than that of CB/PET5 and 2 × 10^3^ times higher than that of Ag/CB/PET5. The Ag-ABCB/PET5 and Ag/CB/PET5 fibers exhibited a rapid increase in electrical conductivity when the filler content was 4–5 wt%. In particular, the Ag-ABCB conductive filler, which was chemically stably bonded, as opposed to the physically-mixed Ag/CB conductive filler, seemed to have a large influence on electrical conductivity improvement.

### 3.3. Morphologies of Conductive Filler/PET Fiber

**Figure 5:**
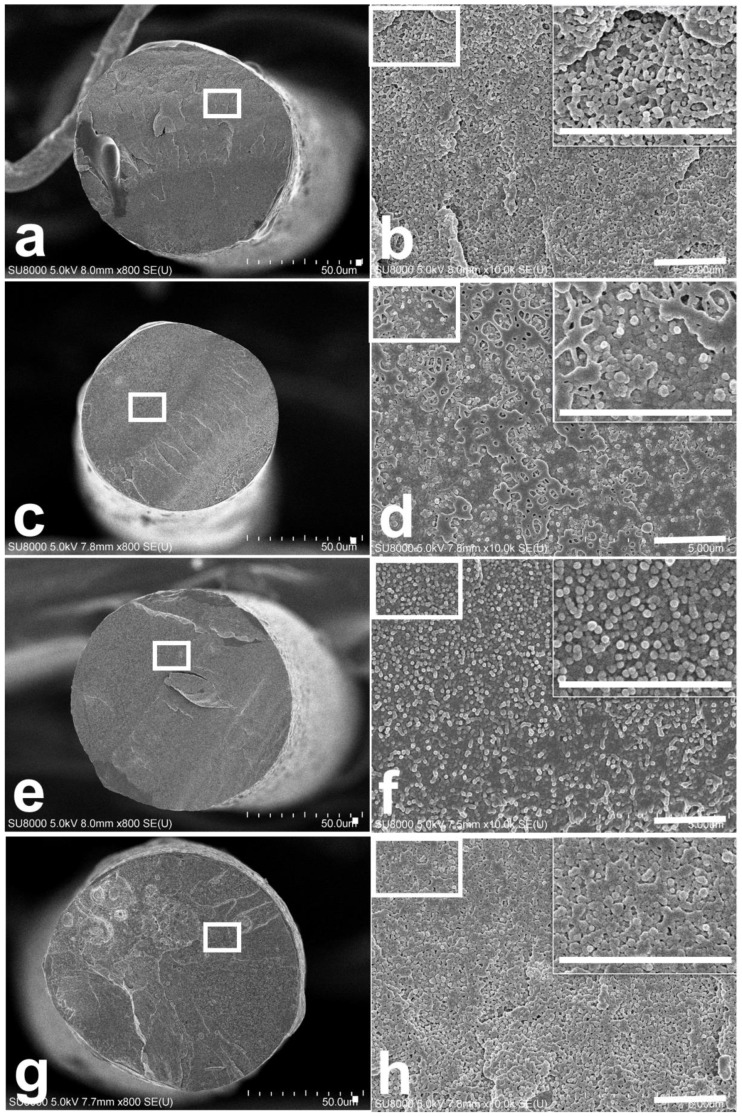
SEM images of (**a**,**b**) CB/PET5; (**c**,**d**) ABCB/PET5; (**e**,**f**) Ag-ABCB/PET5; (**g**,**h**) Ag/CB/PET5. Inset, magnified view of a box of **b**,**d**,**f**, and h. Scale bar is 5 μm.

Electron microscopy was performed to investigate morphology in the PET matrix depending on conductive filler type (Figure 5). The fiber cross section was measured using a conductive filler with a content of 5 wt%, in which the electrical conductivity improved sharply. The diameter of CB/PET5 was 79.3 μm (Figure 5a), ABCB/PET5 was 69.7 μm (Figure 5c), and Ag-ABCB/PET5 was 76.2 μm (Figure 5g). To observe the cross section of each fiber in detail, the white square box portion (Figure 5) was magnified and measured. Results showed high electron mobility in the hexagonal layer plane of the CB crystals, indicating electrical conductivity. The electrical conductivity of CB increased as the particle size decreased, and exhibited a high structure. In other words, fibers using CB and Ag/CB as conductive fillers displayed small CB particles embedded in the polymer, but the CB particle size increased after the functionalization with ABCB, and thus CB was connected (Inset of Figure 5b,d,h). When the Ag-ABCB conductive filler was used, the conductive filler size was larger than that of pristine CB (Inset of Figure 5f) and the contact between the Ag-ABCB performed well. These results strongly support the idea that the Ag-ABCB/PET fiber exhibits better electrical conductivity than the CB/PET and Ag/CB/PET fibers.

## 4. Conclusions

A 4-aminobenzoyl group was functionalized on a CB surface in a PPA/P_2_O_5_ reaction medium, through the “direct” Friedel–Crafts acylation reaction. Ag-ABCB was prepared using reduced AgNO_3_ by NaBH_4_. The morphologies of ABCB and Ag-ABCB were confirmed using SEM images. The ABCB nanocomposite exhibited high structure following functionalization with the 4-aminobenzoyl group on CB, and CB particle size increased with good structure. The Ag nanoparticles were confirmed after loading Ag particles onto the ABCB surface. EA, FT-IR, TGA, XRD and XPS analyses were used to characterize ABCB and Ag-ABCB. The electrical conductivities of Ag-ABCB/PET5 fibers were 8.41 × 10^−8^ S/cm, approximately 3 × 10^4^ and 2 × 10^3^ times higher than that of CB/PET5 and Ag/CB/PET5, respectively. The chemically functionalized Ag-ABCB was much more stable than mechanically-mixed Ag/CB, and Ag particles played a major role in enhancing the electric conductivity. Overall, results indicated that our newly developed Ag-ABCB conductive filler could be used as a conductive fiber. It is expected to be used in a variety of conductive fiber applications, such as antistatic materials and sensors.

## Figures and Tables

**Table 1:** Sample preparation.

Filler^1^	PET	Temperature (°C)						Screw Speed (RPM)	Feeder Speed (RPM)	Winder Speed (MPM)
		Zone #1	Zone #2	Zone #3	Zone #4	Zone #5	Zone #6			
0.5	99.5	200	230	260	260	260	260	110	0.80	800
1.0	99.0	200	230	260	260	260	260	110	0.80	800
2.0	98.0	200	230	260	260	260	260	110	0.80	700
3.0	97.0	200	230	260	260	260	260	110	0.80	-
4.0	96.0	200	230	260	260	260	260	110	0.80	-
5.0	95.0	200	230	260	260	260	260	110	0.80	-

^1^ Filler is CB or ABCB or Ag-ABCB or Ag/CB.
